# Association between STK11 Gene Polymorphisms and Coronary Artery Disease in Type 2 Diabetes in Han Population in China

**DOI:** 10.1155/2017/6297087

**Published:** 2017-02-28

**Authors:** Xiaowei Ma, Ge Bai, Difei Lu, Linjuan Huang, Jianwei Zhang, Ruifen Deng, Shan Ding, Nan Gu, Xiaohui Guo

**Affiliations:** Department of Endocrinology, Peking University First Hospital, Beijing, China

## Abstract

*Background*. Recent studies indicated that the Serine threonine kinase 11 (STK11), which is a key regulator of the AMP-activated protein kinase (AMPK), plays a crucial role in cardiovascular system. This study aimed to investigate whether genetic variations in the STK11 gene affect the risk of coronary artery disease (CAD) in Chinese type 2 diabetics.* Methods*. 5 haplotype-tagging single nucleotide polymorphisms (SNPs) were selected, and 288 CAD-positive cases and 159 CAD-negative controls with type 2 diabetes were genotyped by polymerase chain reaction-restriction fragment length polymorphism (PCR-RFLP) assay.* Results*. The carriers of minor allele A at rs12977689 had a higher risk of CAD compared to the homozygotes of CC (OR = 1.572, 95% CI = 1.039–2.376, *p* = 0.035), and the difference was still significant after adjustment for the other known CAD risk factors (OR′ = 1.184, 95%  CI′ = 1.036–1.353, *p*′ = 0.013).* Conclusion*. Genetic variability at STK11 locus is associated with CAD risk in type 2 diabetes in the Chinese population.

## 1. Introduction

The rapid increase in prevalence of coronary artery disease has been a major health challenge worldwide, especially in type 2 diabetic patients. A number of studies showed that the diabetic patients had a 2–4-fold greater risk of developing CAD than those of nondiabetics [1]. In addition to the important contribution of environmental factors including changes in dietary patterns and lifestyle, genetic determinants play a major role in the individual susceptibility to coronary artery disease (CAD).

Plenty of prior studies have proved that adiponectin, an adipocyte-secreted adipocytokine, has the antidiabetic and antiatherogenic effects [2–4] partly by evoking nitric oxide (NO) production and endothelium-dependent vasodilatation via activation of AMP-activated protein kinase (AMPK) [5, 6].

Serine threonine kinase 11 (STK11) is a key regulator of a family of kinases including AMPK. It was initially discovered because of its role as a tumor suppressor and mutated in a number of cancers (Peutz-Jeghers Syndrome, lung and cervical cancer) [7]. But it is becoming clear that STK11 has a wide range of biological roles that reach far beyond that. Recently, convincing evidence has been presented that the endothelial nitric oxide synthase (eNOS) activation depends on STK11 mediated by adiponectin pathway [8]. And increasing studies were focused on its crucial role in protecting cardiac muscles from damage during ischemia [9, 10].

However, despite the potential importance of STK11 activity in terms of regulating cardiovascular systems, very few studies had been conducted on STK11 gene and genetic variants in heart diseases, especially in coronary artery disease. We hypothesized that genetic variants in STK11 gene might affect the CAD risk through impacting adiponectin signaling pathway. To confirm the hypothesis, we investigated the CAD-positive and CAD-negative Han patients with type 2 diabetes in China.

## 2. Materials and Methods 

### 2.1. Subjects

The Chinese Han individuals with type 2 diabetes were studied. All patients and controls were recruited among type 2 diabetic patients who underwent cardiac catheterization at Peking University First Hospital (PUFH) from 2007 to 2010. Type 2 diabetes was defined by 1999 World Health Organization criteria [11], which stipulates fasting plasma glucose ≥ 7.0 mmol/L and/or 2-hour plasma glucose ≥ 11.1 mmol/L or casual plasma glucose (random blood sugar) ≥ 11.1 mmol/L. Type 1 diabetic patients and subjects with active inflammatory conditions, autoimmune diseases, malignancies, using of immunosuppressive drugs, and known hematological disorders were excluded from the study.

In agreement with the Helsinki Declaration, all of total 447 unrelated subjects gave informed consent to participate in this study. The CAD-positive cases (*n* = 288) were defined as those who had a stenosis > 50% in at least one major coronary artery or their main branches. The CAD-negative type 2 diabetic patients (*n* = 159) had a coronary stenosis ≤ 50% with cardiac catheterization or spiral computer tomography (CT) coronary angiography [12].

Other demographic data with known cardiovascular risk factors including age, gender, body mass index (BMI), fasting plasma glucose, lipid profile, and the histories of hypertension (≥140/90 mmHg or receiving antihypertensive therapies) and smoking (“ever” or “never,” “ever” defined as having smoked more than 1 cigarette per day for more than 1 year) were collected for all the subjects (Table 1).

### 2.2. Ethical Approval of the Study Protocol

The study protocol and informed consent procedures were approved by the research ethics committees of PUFH.

### 2.3. Genotyping

Genomic DNA was extracted from leukocytes obtained from 2 mL EDTA-coated peripheral blood samples. All 5 haplotype-tagging SNPs at STK11 locus with 10 exons spanning 21 kb on chromosome 19p13.3 were selected in this study from CHB data in HapMap Phase II database R#27 (https://www.hapmap.org) (*r*^2^ < 0.8, MAF ≥ 0.05), including rs6510599 (C>T), rs35369365 (A>G), rs9282860 (C>T), rs3764640 (G>T), and rs12977689 (A>C). All of these 5 SNPs are located in introns.

These 5 SNPs were typed by polymerase chain reaction-restriction fragment length polymorphism (PCR-RFLP) assay with the genotyping success rates of 85%–95% and repeatability rates of 98%–100%. Directly DNA sequencing was used to further confirm the genotypes for each SNP for 1 percent of the cases and controls with the concordance rates between RFLP and DNA sequencing of average 93.3%.

### 2.4. Statistical Analysis

The associations between CAD and genotypes were analyzed by a *χ*^2^ test and multiple stepwise regression analysis was utilized to adjust for potential confounders. The clinical and laboratory data were expressed as mean ± SD or percentage. Qualitative variables were compared using *χ*^2^ test, and quantitative variables using independent samples *t* test. The Hardy-Weinberg equation was employed to determine the proportion of each genotype. Adjusted odd ratios (ORs) and 95% confidence intervals (95% CIs) were calculated. The null hypothesis was rejected when *p* was considered significant at <0.05. Statistical analysis was performed using the SPSS software (version 16.0 for windows, USA). Haploview (version 4.2, USA) was used for haplotype analysis. Haplotype frequencies were analyzed by *χ*^2^ test and haplotype block was defined by confidence interval method in the software.

## 3. Results

### 3.1. Clinical Characteristics

Comparing with the control group, patients with CAD were more likely to be men or smokers. Lower plasma lipids levels were found in CAD group, which may be due to the fact that the clinical laboratory profile was after clinical intervention (Table 1).

### 3.2. Genotype Distributions

No significant deviation from the Hardy-Weinberg equilibrium was observed in controls at all 5 SNPs (*p* > 0.05).

All the 5 SNPs had similar allele frequencies in the two groups (Table 2). At rs12977689, the carriers of major allele A (AC+AA) had a 1.5-fold increased CAD risk compared to homozygotes of CC (OR = 1.572, 95% CI = 1.039–2.376, *p* = 0.035). After adjustment for the other known cardiovascular risk factors (age, gender, duration of diabetes, BMI, plasma lipid profile, smoking, and hypertension status), the trend was consistent (adjusted OR′ = 1.184, 95%  CI′ = 1.036–1.353, *p*′ = 0.013) (Table 3). At another SNP rs35369365, there was a significant association of AG genotype with CAD, revealing a 1.8-fold higher risk (adjusted OR′ = 1.848, 95%  CI′ = 1.056–3.236, *p*′ = 0.032). In comparison with the carriers of AA genotype, those carrying minor allele G (AG+GG) had the tendency of an increase in CAD risk after adjustment for the known cofounders (*p*′ = 0.069) (Table 3).

### 3.3. Haplotype Analysis

**Figure 1:**
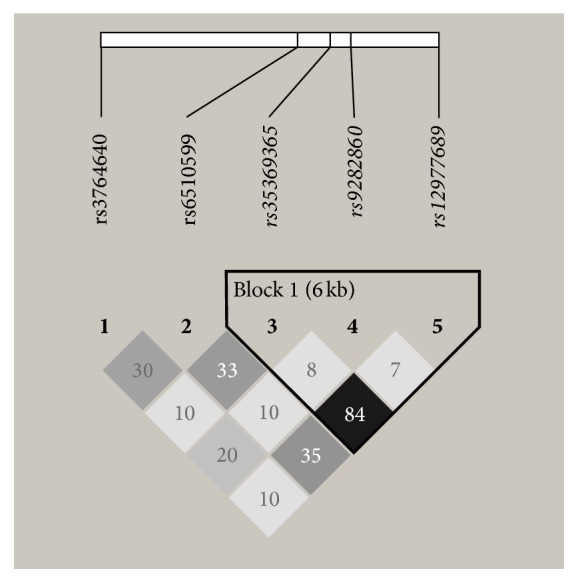
The *r*^2^ values and the linkage disequilibrium (LD) plot between SNPs at STK11 locus. The color of the squares indicated the intensity of the linkage between SNPs. The *r*^2^ values were showed in the squares. The triangular block circled the LD plot (confidence interval method).

The *r*^2^ values and the linkage disequilibrium (LD) plot between SNPs of all patients in the study population were shown and 3 out of 5 SNPs were in a LD block (Figure 1). Of all the haplotypes constructed by rs35369365, rs9282860, and rs12977689 in an order of their physical positions, there were total 4 common haplotypes with frequencies of >5%. But none of the 4 common haplotypes at STK11 gene was associated with CAD risk in the type 2 diabetic patients (Table 4).

### 3.4. SNP rs12977689 and BMI

In further analysis, we found SNP rs12977689 may also affect BMI. The carriers of risk allele A at rs12977689 had a tendency of a higher BMI level compared to homozygotes of CC (*p*′ = 0.060) (Table 5).

## 4. Discussion

The kinase STK11 is required for activation of AMP-activated protein kinase. It normally functions in a large protein complex including pseudokinase STRAD (Serine/threonine protein kinase 20 related adaptor) and the scaffolding MO25 (mouse protein 25) [13]. Under basal conditions, STK11 is localized in the nucleus while APPL1 (adaptor protein containing PH domain, PTB domain, and leucine zipper motif 1) and inactive AMPK in the cytosol. Adiponectin induces the translocation of STK11 from the nucleus into cytosol in association with the binding of APPL1 to AdipoR (the membrane receptor of adiponectin), followed by the phosphorylation of AMPK [14, 15] at a specific threonine residue (Thr172) on *α* subunit [16, 17].

The STK11-dependent AMPK activation could block IKK-NF-*κ*B signaling; thus it could inhibit proinflammatory cytokines and adhesion molecules production and also reduce interleukin-18-mediated endothelial cell death [18]. Adiponectin-stimulated STK11-AMPK signaling results in enhancement of eNOS and in turn, production of nitric oxide is increased which could cause the promotion of vasodilation, inhibition of platelet aggregation, and proliferation of vascular smooth muscle [19]. In addition, STK11-AMPK phosphorylation inactivates acetyl-CoA carboxylase, resulting in fatty acid oxidation [20]. In general, since STK11 has been shown to be an activator of AMPK as above, it may indirectly contribute to a major part of the cellular effects of AMPK and therefore plays a crucial role in antiatherosclerosis and coronary protection.

Recently, accumulating studies focused on the physiological function of STK11 in cardiovascular system. It was demonstrated that STK11 is critical in maintaining normal cardiac function under aerobic condition or during recovery after ischemia [9]. After ischemia reperfusion, dominant-negative AMPK overexpression inhibited cardiac function through the suppression of glucose uptake and fatty acid *β*-oxidation in cardiac myocytes [20–22]. Also, the decrease of AMPK leads to the reduction of subsequent endothelial NO production and further impairs endothelium-based vasodilation. Conversely, Italian researchers found that myocardial perfusion in chronic diabetic mice was improved by the upregulation of STK11 and AMPK signaling [23]. This evidence indicated STK11 might also contribute to the myocardial perfusion in a diabetic model.

Besides, STK11 appears to have more biological effects in cardiovascular system other than catalyzing AMPK. Interestingly, the study on STK11 knock-out mice showed that the mice exhibited cardiac hypertrophy and dysfunction with a decrease in capillary density of both atria and ventricles, which was consistent with significant lower mRNA and protein levels of vascular endothelial growth factor [24]. Similarly, a report from United Kingdom indicated that deletion of STK11 in the endothelial lining of blood vessels induced a decline in TGF*β* (transforming growth factor *β*) production which was activated independent of AMPK by STK11 and resulted in fragile and distorted vessels characterized by loss of the supporting vascular smooth muscle cells which were necessary for stabilization and maturation [25]. The above evidence indirectly implies that STK11 may contribute to individual susceptibility to angiogenesis, and this effect is independent of AMPK activation. The mechanism could be explained that the endothelial cells were relying on TGF*β*-based communication between endothelium and smooth muscles to recruit smooth muscle cells into the newly formed blood vessels. And the role of STK11 in this process appeared to regulate TGF*β* availability to the nearby smooth muscle and mesenchymal cells [7]. Taken together, changes in the expression of STK11 gene or, in other words, in its functions may affect cardiac and vascular endothelial function.

So we postulated that the genetic variability in the STK11 gene could affect the susceptibility to CAD in type 2 diabetic patients in China. To test the hypothesis, we carried out a case-control study to assess the potential effect of STK11 gene on CAD in Chinese Han population with type 2 diabetes. We carefully designed and implemented this study. First, we recruited subjects from type 2 diabetes patients as the CAD as the diabetic population have a higher morbidity and mortality of CAD, and it is clinically important to screen and to prevent this microvascular complication at the early stage. Second, we carefully selected the CAD cases by using a widely accepted criterion. And moreover, we used HapMap website to choose tagging SNPs and these 5 SNPs in our study covered 100% of the STK11 gene. Finally, to minimize confounding factors, we performed logistic regression to adjust age, BMI, sex, duration of diabetes and hypertension, smoking, and plasma lipid status.

In this study, we found that there was an association between SNPs at STK11 locus and individual susceptibility to CAD in the Chinese type 2 diabetes mellitus (DM) patients. The risk of CAD for the carriers of allele A of rs12977689 was 1.6 times as high as for the noncarriers (*p* = 0.035). And the association after adjustment was consistent, which suggested that rs12977689 might influence the CAD risk independent of the other known cardiovascular risk factors in the Chinese Han type 2 DM patients. Meanwhile, rs35369365 at STK11 locus might be also associated with CAD susceptibility in the subjects with type 2 DM, with borderline statistical significance after adjustment. As of date, none of the studies on genetic variations at STK11 locus was reported among CAD subjects with or even without type 2 DM.

STK11 plays a key role in cellular metabolism. In the adiponectin pathway, the phosphorylation of AMPK by STK11 leads to inactivation of acetyl CoA carboxylase, resulting in fatty acid oxidation [26]. Furthermore, STK11 gene has been demonstrated to have a relationship with insulin sensitivity [27].

As a result, to investigate the mechanism of the STK11 gene influencing the CAD morbidity, we assessed the association between the SNP rs12977689 and status of BMI, fasting plasma glucose, lipid profile, and the histories of hypertension. As we have found, there may also be an association between SNP rs12977689 and body mass index, with a borderline significant difference, which may indirectly imply that genetic variability of STK11 gene may affect the individual susceptibility to CAD via regulating metabolism.

Although the SNPs we found associated with CAD risk in the study population are all placed in intronic regions, it is already known that intronic SNPs may regulate gene expression and alternative splicing [28, 29]. Therefore, intron SNPs may affect the expression levels of STK11, influencing the AMPK activation and affecting the angiogenesis of the myocardium. In addition, it seems more likely that these SNPs rs12977689 and rs35369365 are genetic markers which may be in high linkage disequilibrium with neighboring functional SNPs in the kinase gene region, thereby potentially affecting the structure and/or catalytic activity of the enzyme.

The studied subjects were the diabetic patients who took cardiac catheterization or spiral CT coronary angiography procedure in PUFH. Multiple studies confirmed the high correlation of the spiral CT coronary angiography with cardiac catheterization, a gold standard, for CAD diagnosis, with its specificity of 95%  ± 3.9%, sensitivity of 82.3%  ± 3.4%, false-negative rate at 5%  ± 4.3%, and false-positive rate at 7.9%  ± 6.7% [30]. As of spiral CT for CAD diagnosis in PUFH, it had the specificity of 97%, sensitivity of 73–80%, false-negative rate at 2.6%, and false-positive rate at 5.9% [12]. Therefore, in order to minimize the effect that the recruited subjects alone incur in the case-control study, CAD-positive group was comprised of the diabetic patients with CAD diagnosed only based on cardiac catheterization, and the control group was defined on either cardiac catheterization or spiral CT coronary angiography.

We are conscious that there are some limitations in this study. The sample size was not large enough, and thus the statistical power for some SNPs with lower frequencies of minor allele was not strong enough to make negative conclusions. The clinical features were not perfectly matched between the case and control groups, which might bias the results. And further function study should be completed on the genetic variants at STK11 locus.

This study only provided preliminary evidence about the association of genetic variations of STK11 with development of CAD in T2DM subjects in China. Further studies should be done to confirm the findings in a large patient series and even a number of ethnics and races. A larger number of the patients should be continuously recruited to reduce the variations. The mechanisms by which the genetic variability in the gene contributes to the CAD risk should be further investigated. Also, our findings should be confirmed in prospective studies before STK11 polymorphisms can be applied to predict the risk of CAD in type 2 DM patients in the Chinese Han population.

## Figures and Tables

**Table 1:** Characteristics of study population.

Group (number)	CAD (288)	Control (159)	*p* value
Male (%)	65.2	45.2	0.000^*∗*^
Age (y)	63.78 ± 9.10	62.08 ± 10.08	0.070
DM duration (y)	8.18 ± 7.31	8.29 ± 6.47	0.878
Hypertension (%)	77.8	71.2	0.150
BMI (kg/m^2^)	25.95 ± 3.40	25.77 ± 3.84	0.640
HbA1c (%)	7.36 ± 1.50	7.12 ± 1.47	0.193
FBG (mmol/L)	7.23 ± 2.56	6.79 ± 1.86	0.074
TG (mmol/L)	1.62 ± 0.94	1.81 ± 1.32	0.105
TCHO (mmol/L)	4.09 ± 1.02	4.55 ± 0.95	0.000^*∗*^
HDL (mmol/L)	0.98 ± 0.29	1.09 ± 0.33	0.001
LDL (mmol/L)	2.47 ± 0.84	2.71 ± 0.82	0.009^*∗*^
Smokers (%)	49.3	35.2	0.010^*∗*^

TG, triglycerides; TC, total cholesterol; LDL-C, low density lipoprotein-cholesterol; HDL-C, high density lipoprotein-cholesterol.

Continuous variables were expressed as mean ± standard deviation. *p* values of continuous variables were calculated by independent samples *t*-test.

Categorical variables were expressed as percentage. *p* values were obtained by *χ*^2^ test. *∗* refers to the significant difference between the CAD and control groups.

**Table 2:** Distributions of alleles at STK11 in CAD-positive and CAD-negative subjects.

SNPs	Alleles	CAD	Control	OR	95% CI	*p*
		*n* (%)	*n* (%)			
rs6510599	C	324 (59.3)	157 (56.5)	1.125	0.840–1.507	0.455
	T	222 (40.7)	121 (43.5)			
rs35369365	G	182 (35.5)	96 (32.8)	1.172	0.866–1.586	0.320
	A	330 (64.5)	204 (67.2)			
rs9282860	T	76 (13.7)	40 (13.9)	1.014	0.672–1.532	1.000
	C	478 (86.3)	248 (86.1)			
rs3764640	G	176 (35.9)	104 (38.2)	1.104	0.813–1.500	0.531
	T	314 (64.1)	168 (61.8)			
rs12977689	C	341 (65.1)	199 (70.6)	1.287	0.941–1.759	0.117
	A	183 (34.9)	83 (29.4)			

**Table 3:** Association between CAD and SNPs at STK11 locus in different genetic model.

SNPs	Genotype	CAD	Control	OR	95% CI	*p*	OR′	95% CI′	*p*′
		*n* = 288	*n* = 159						
rs3764640	TT	98 (40.0)	53 (39.0)	1.155	0.660–2.020	0.665	1.019	0.445–2.331	0.964
	GG+GT	147 (60.0)	83 (61.0)	1			1		
rs6510599	CC+CT	233 (85.3)	116 (83.5)	1.055	0.680–1.603	0.913	1.104	0.840–1.452	0.477
	TT	40 (14.7)	23 (16.5)	1			1		
rs35369365	GG+GA	160 (62.5)	83 (55.0)	1.365	0.908–2.054	0.144	1.275	0.981–1.656	0.069
	AA	96 (37.5)	68 (45.0)	1			1		
rs9282860	CC	204 (73.6)	105 (72.9)	1.038	0.659–1.635	0.908	1.002	0.965–1.040	0.926
	TT+TC	73 (26.4)	39 (27.1)	1			1		
rs12977689	AA+AC	161 (61.5)	71 (50.4)	1.572	1.039–2.376	0.035^*∗*^	1.184	1.036–1.353	0.013^*∗*^
	CC	101 (38.5)	70 (49.6)	1					

OR′, 95%  CI′, *p*′: After adjustment for age, sex, duration of diabetes and hypertension, BMI, smoking, and plasma lipid status. *∗* refers to the significant difference between the CAD and control groups.

**Table 4:** Haplotype analyses in patients with CAD and control subjects.

SNPs	Haplotype	Frequency		*p*
	1 2 3	CAD	Control	
	A C C	0.505	0.524	0.585
1: rs35369365				
	G C A	0.342	0.300	0.217
2: rs9282860				
	A T C	0.123	0.128	0.827
3: rs12977689				
	G C C	0.019	0.036	0.138

Haplotypes with frequency > 0.05 were estimated using Haploview software.

*p* values were calculated by chi-squared test.

**Table 5:** Characteristics of different genotype at rs12977689.

Genotype (number)	AA+AC (232)	CC (171)	*p* value
BMI (kg/m^2^)	26.27 ± 3.36	25.58 ± 3.59	0.060
HbA1c (%)	7.36 ± 1.56	7.29 ± 1.41	0.732
FBG (mmol/L)	7.14 ± 2.28	7.23 ± 2.66	0.725
TG (mmol/L)	1.67 ± 1.00	1.77 ± 1.21	0.405
TCHO (mmol/L)	4.20 ± 1.08	4.33 ± 0.97	0.252
HDL (mmol/L)	1.02 ± 0.31	1.03 ± 0.33	0.832
LDL (mmol/L)	2.49 ± 0.81	2.63 ± 0.90	0.145
Hypertension (%)	77.5	73.9	0.468

TG, triglycerides; TC, total cholesterol; LDL-C, low density lipoprotein-cholesterol; HDL-C, high density lipoprotein-cholesterol.

Continuous variables were expressed as mean ± standard deviation. *p* values of continuous variables were calculated by independent samples *t*-test.

Categorical variables were expressed as percentage. *p* values were obtained by *χ*^2^ test.

## Abbreviations

STK11: Serine threonine kinase 11
AMPK: AMP-activated protein kinase
CAD: Coronary artery disease
SNP: Single nucleotide polymorphism
PCR-RFLP: Polymerase chain reaction-restriction fragment length polymorphism
NO: Nitric oxide
eNOS: Endothelial nitric oxide synthase
PUFH: Peking University First Hospital
CT: Computer tomography
BMI: Body mass index
DNA: Deoxyribonucleic acid
EDTA: Ethylenediaminetetraacetic acid
MAF: Minor allele frequency
OR: Odd ratio
CI: Confidence interval
LD: Linkage disequilibrium
STRAD: Serine/threonine protein kinase 20 related adaptor
MO25: Mouse protein 25
APPL1: Adaptor protein containing PH domain, PTB domain, and leucine zipper motif 1
IKK: I*κ*B kinase
NF-*κ*B: Nuclear factor *κ*B
TGF*β*: Transforming growth factor *β*
DM: Diabetes mellitus.
